# Stability of Standard Electrolytic Conductivity Solutions in Glass Containers

**DOI:** 10.6028/jres.107.032

**Published:** 2002-10-01

**Authors:** Rubina H. Shreiner

**Affiliations:** National Institute of Standards and Technology, Gaithersburg, MD 20899-8393

**Keywords:** conductivity, containers, packaging, potassium chloride, stability, standards

## Abstract

The stability of solutions having an electrolytic conductivity, ***κ***, of 5 μS/cm to 100 000 μS/cm packaged in glass screw-cap bottles, glass serum bottles, and glass ampoules was monitored for 1 year to 2 years. The conductivity was determined by measuring the ac resistance of the solution. Mass loss was also monitored for solutions packaged in bottles. The solutions were prepared using KCl in water (***κ*** ≥100 μS/cm) or KCl in 30 % (by mass) n-propanol 70 % (by mass) water (***κ*** ≤ 15 μS/cm). The conductivity changes were compared by packaging type and by nominal ***κ***. The main causes of the ***κ*** changes are evaporation (screw-cap bottles) and leaching (screw-cap bottles, serum bottles, and ampoules). Evaporation is determined from mass loss data; leaching occurs from the glass container with no change in mass. The choice of optimal packaging, which depends on the conductivity level, is the packaging in which ***κ*** changes the least with time. Ampoules are the most suitable packaging for standards having nominal ***κ*** values of 500 μS/cm to 100 000 μS/cm. Screw-cap bottles are most suitable for standards having a nominal ***κ*** of 5 μS/cm to 100 μS/cm.

## 1. Introduction

The measurement of electrolytic conductivity, ***κ***, is used to monitor the ionic content of solutions (e.g., fruit juices, soft drinks, dialysis fluid, and natural waters) and the purity of water (e.g., drinking water, wastewater, process water). Many industries, including pharmaceutical, power, and health care, rely on electrolytic conductivity standards to calibrate electrolytic conductivity meters and cells. The availability of standards with accurate and stable ***κ*** values is crucial to those industries.

The monitoring equipment is calibrated by measuring the resistance of a standard, *R*_c_, having a known conductivity, ***κ***_c_, in a conductivity cell. The cell constant, *K*_cell_, is then determined by Eq. (1),

$$K_{\text{cell}}=\kappa_{c}R_{c}.$$ (1)

The accuracy of this calibration, and the subsequent measurements, is determined by the accuracy of the standard. The conductivity of a solution, ***κ***_s_, can then be determined by Eq. (2),

$$\kappa_{s}=K_{\text{cell}}/R_{s}$$ (2)

where *R*_s_ is the resistance of the solution measured in a cell with a known *K*_cell_.

A practical consideration in the accuracy of electrolytic conductivity standards, as with all standards, is their stability, or change in certified value versus time. Although there is a large body of data with regard to standard electrolytic conductivity solutions, e.g., Refs. [1–10], data regarding the long-term stability of the standard solutions are lacking. Obviously, any change from the certified value will compromise the accuracy of the standard at the time of use and must be considered in establishing both the uncertainty in ***κ*** and the expiration date of the reference material. This paper reports the change of ***κ*** in solutions packaged in a variety of container types observed for several years.

NIST prepares and certifies electrolytic conductivity standards in the range of 5 μS/cm to 100 000 μS/cm as SRMs (Standard Reference Materials) 3190 to 3199. The certificates for these SRMs typically expire in 1 year to 2 years because of the difficulties in maintaining their long-term stability. Stability is one of the factors in the certified uncertainties, which vary from 0.07 % to 4 % in the most recent certifications of the highest and lowest conductivity standards in this group, respectively. Neglecting the contribution of instability, the certified uncertainties of these SRMs would be in the range of 0.07 % to 2 % (Table 1). The goal of this study is to achieve an uncertainty close to the target values for each SRM listed in Table 1.

All of the containers in this study were glass. Evaporation and leaching are the two main problems with glass containers. Evaporation occurs through the space between the cap and the bottle, causing a simultaneous mass loss and ***κ*** increase of the solution. The ***κ*** increase would be approximately proportional to mass loss from evaporation. A ***κ*** increase of the solution with no mass loss would be indicative of an increase in the ionic strength from leaching. Three types of packaging have been tested: glass screw-cap bottles, glass serum bottles, and glass ampoules. The stability of solutions ranging in conductivity from 5 μS/cm to 100 000 μS/cm was monitored. The causes of instability and the choice of the best packaging type are discussed.

## 2. Experimental

### 2.1 Solution Preparation and Packaging

Containers that were readily available to users and producers of conductivity standards were chosen for this study. Screw-cap bottles, serum bottles, and ampoules were purchased commercially and were each made of borosilicate glass [11, 12]^1^. The 500 mL screw-cap bottles had polypropylene plug seal caps. The 100 mL serum bottles had aluminum caps, which were lined with Teflon^®^ faced gray butyl septa. The 50 mL ampoules were sealed in a natural gas/O_2_ flame. The glass composition of the screw-cap bottles and the ampoules was significantly different from the glass composition of the serum bottles (Table 2). Thus, three parameters differed among the bottles studied: closure type, glass type, and volume to surface area ratio.

Solutions were prepared using deionized water (***κ*** < 0.06 μS/cm at delivery) and potassium chloride (reagent grade). Mixed aqueous-nonaqueous solutions (5 μS/cm and 15 μS/cm, only) were prepared using n-propanol (assayed by the manufacturer at 100 %) and deionized water. A total of fifteen solutions were separately prepared and monitored. The solutions’ nominal conductivities, the packaging types, and the approximate masses of KCl are displayed in Table 1. The solutions were thoroughly mixed and homogenized. All solutions were equilibrated with atmospheric CO_2_ prior to packaging.

Each screw-cap bottle was filled with ≈ 500 mL of solution and immediately capped. The cap-bottle juncture of the screw-cap bottles used for the 100 μS/cm and 1000 μS/cm solutions were wrapped in Parafilm (44 d after bottling for the 1000 μS/cm solution; the day of bottling for the 100 μS/cm solution). Each serum bottle was filled with ≈ 100 mL of solution and immediately capped. The mass loss of each screw-cap bottle and serum bottle was monitored. Each ampoule was filled with ≈ 50 mL of solution (air head-space) and immediately sealed in a natural gas/O_2_ flame. All of the containers for a given solution were filled and capped (or sealed) in 1 day. The mass loss of the 1000 μS/cm solution packaged in ampoules was monitored for the first 23 d. Ampoules that lost mass were discarded, since mass loss indicated a pinhole. For the other solutions packaged in ampoules, a vacuum was pulled on the seal of each ampoule. If there were a hole in the seal of the ampoule, liquid would be visible in the tubing. Any ampoule in which liquid was observed in the tubing was discarded.

All of the containers were stored on shelves and/or in boxes in rooms with an air temperature of 20 °C ± 5 °C.

Over the 2 year study, random units were selected for measurement and each unit was only used once. A “unit” refers to the set of containers (1 screw-cap bottle, 2 to 3 serum bottles, or 5 to 6 ampoules) needed to obtain a sufficient quantity of solution to perform one measurement (including necessary preliminary rinses of the cell). Three to seven units were measured at each time period and the mean of these measurements was taken as the value of the solution at that time.

### 2.2 Equipment and Measurement Method

The equipment used for the measurements has been previously described [13]. An ac measurement technique was used to determine the conductivity of each unit at 25.000 °C ± 0.003 °C [13]. The conductivity cells were calibrated with primary standards [4, 7, 13]. The cell was rinsed 4 to 5 times and filled with solution from the same unit. The ac resistance was measured at 1 kHz (*R*_1 kHz_) and 2 kHz (*R*_2 kHz_) and the resistance was extrapolated linearly to infinite frequency [14]. The lead resistance was subtracted from the extrapolated resistance to obtain the resistance of the solution, *R*. The conductivity, ***κ***, of the solution was calculated by Eq. (2).

## 3. Results and Discussion

The results of this study were obtained by grouping the data according to the type of container and, separately, by the ***κ*** value. Thus, the following discussion is organized accordingly.

### 3.1 Comparison by Container

**Fig. 1:**
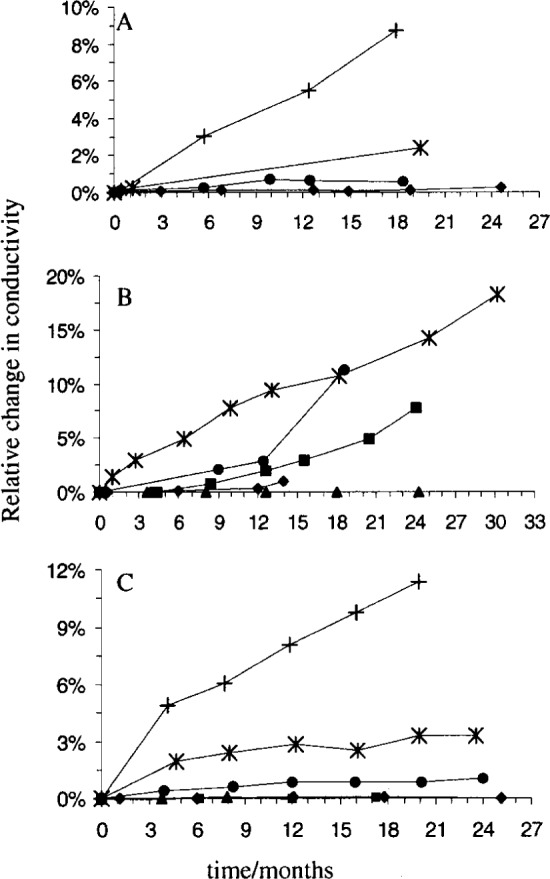
Solution instability by packaging type. A—screw-capped bottles; B—serum bottles; C—ampoules; + 5 μS/cm, ✵ 15 μS/cm, ● 100 μS/cm, ■ 500 μS/cm, ♦ 1000 μS/cm, ▲ 100 000 μS/cm. The standard deviation for each point is given in Tables 3 to 5.

The mean conductivities determined at each time period are shown in Fig. 1 and the standard deviations are shown in Tables 3–5. The relative change in ***κ*** with time increases with decreasing ***κ*** for all three packaging types. The conductivity data for each solution illustrates consistent batch change and not the change of an individual unit, since each unit was measured only once.

A ***κ*** increase (Fig. 1A) and mass loss, generally < 0.5 %, were observed for solutions packaged in the screw-cap bottles. The mass loss is caused by evaporation. Upon closer inspection of the bottles, it was noticed that the caps had loosened and needed to be retightened. Previous to these experiments, screw-cap bottles occasionally leaked during shipment, further supporting the hypothesis of an imperfect cap. The relative change in mass due to evaporation is approximately equal to the relative ***κ*** change if evaporation is the only cause of the solution’s instability. For the 1000 μS/cm solution, the entire relative ***κ*** change (0.25 %) may be explained by mass loss. However, the relative ***κ*** changes for the 5 μS/cm (≈ 5.5 %), 15 μS/cm (≈ 2.4 %) and 100 μS/cm (≈ 0.7 %) solutions were significantly greater than the relative mass loss. The disparity between the ***κ*** data and the mass loss data indicates that leaching from the glass, yielding ions, especially sodium [15–17], to the solution, must also be occurring. Both leaching and evaporation contributed to the observed ***κ*** change in the 5 μS/cm, 15 μS/cm, and 100 μS/cm solutions.

A ***κ*** increase for solutions with ***κ*** ≤ 1000 μS/cm (Fig. 1B) and mass loss, generally < 0.07 %, were observed for the serum bottles. This mass loss is caused by evaporation. The conductivity of the 100 000 μS/cm solution did not change significantly during the time it was monitored. However, the relative ***κ*** change for the 15 μS/cm (≈ 18 %), 100 μS/cm (≈ 11 %), 500 μS/cm (≈ 8 %), and 1000 μS/cm (≈ 1 %) solutions was significantly larger than the observed mass loss. The disparity between the relative ***κ*** change and relative mass loss indicates that leaching from the glass is occurring. Leaching is the major cause of ***κ*** change for these solutions. The serum bottles have a 1.6 times smaller volume to surface area ratio than the screw-cap bottles. Also, the glass used for the serum bottles has a higher concentration of leachable species than the glass used for the screw-cap bottles or the ampoules (Table 2). Therefore, leaching from the serum bottles would result in a higher concentration of ions in the solution and would increase the conductivity of the solution to a greater extent than in the screw-cap bottles, as observed.

A ***κ*** increase was observed for solutions with ***κ*** ≤ 100 μS/cm (Fig. 1C) packaged in ampoules, but a mass loss was not observed. Evaporation was eliminated. The conductivity increases that were observed for the 5 μS/cm (≈ 11 %), 15 μS/cm (≈ 3 %), and 100 μS/cm (≈ 0.9 %) solutions are due to leaching. However, leaching did not affect the conductivities of the 500 μS/cm, 1000 μS/cm, and 100 000 μS/cm solutions, which did not change significantly. Ampoules have a 1.6 times smaller volume to surface area ratio than the serum bottles. However, the concentration of leachable species in the glass is less in the case of ampoules than with serum bottles (Table 2). Therefore, it is not surprising that the leaching effect observed with ampoules is less than the leaching effect observed with serum bottles.

The scatter in ***κ*** of the 3 to 7 units measured at each time period increased with time for solutions that had increases in mean ***κ*** with time. The increase in standard deviation with increasing time, shown in Tables 3 to 5, is indicative of an increase in scatter. Variations in evaporation and/or leaching would cause the observed bottle-to-bottle or ampoule-to-ampoule differences. The effects of evaporation or leaching are slightly different for each container and are expected to increase with time. For some solutions, the scatter initially increased with time then appeared to level off. A bottle-to-bottle or ampoule-to-ampoule effect was observed for solutions that had no conductivity change with time, but no significant increase in the scatter with time was observed.

### 3.2 Comparison by Conductivity

**Fig. 2:**
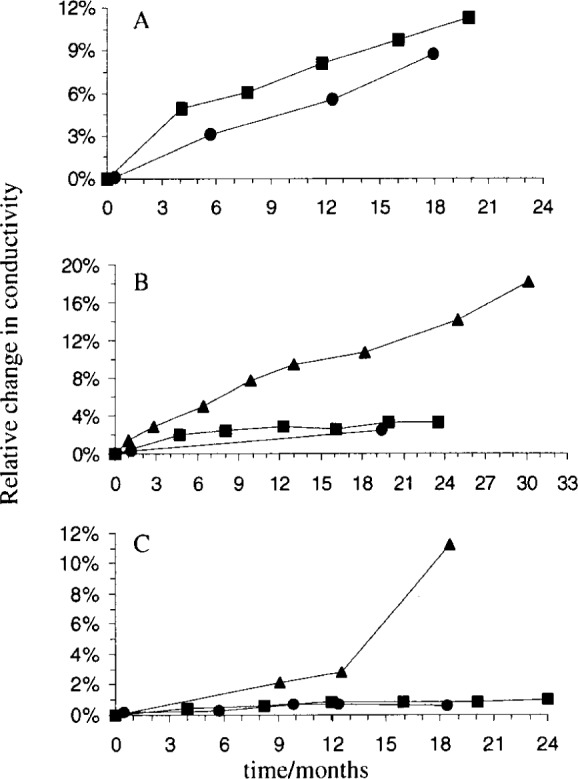
Change in conductivity of the 5 μS/cm, 15 μS/cm, and 100 μS/cm solutions. A—5 μS/cm; B—15 μS/cm; C—100 μS/cm; ● screw-capped bottles, ▲ serum bottles, ■ ampoules. The standard deviation for each point is given in Tables 3 to 5.

**Fig. 3:**
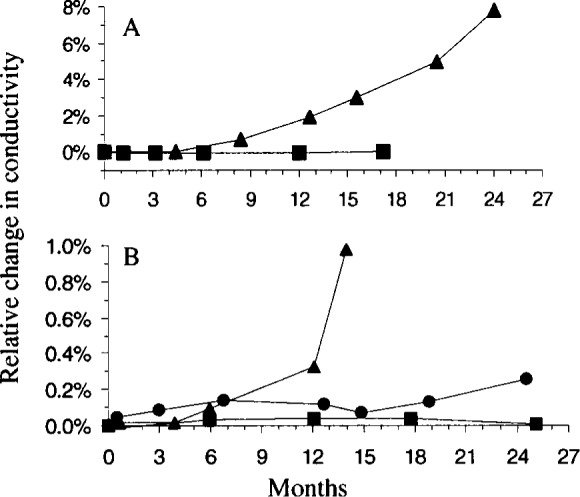
Change in conductivity of the 500 μS/cm and 1000 μS/cm solutions. A—500 μS/cm; B—1000 μS/cm; ● screw-capped bottles, ▲ serum bottles, ■ ampoules. The standard deviation for each point is given in Tables 3 to 5.

The data are reorganized according to levels of conductivity in Figs. 2 and 3. The 5 μS/cm solution was most stable when packaged in screw-cap bottles (Fig. 2A). The 15 μS/cm solution was similarly stable in screw-cap bottles and ampoules (Fig. 2B). The 100 μS/cm solution was similarly stable in screw-cap bottles and ampoules (Fig. 2C). The 500 μS/cm and 1000 μS/cm solutions were most stable in ampoules (Fig. 3). The 100 000 μS/cm solution had no measurable change in conductivity in either the serum bottles or the ampoules and is not included in the graphs.

## 4. Conclusions

The experiment indicates that leaching, which is dependent upon glass type, is the dominant source of instability for the low-conductivity solutions (***κ*** ≤ 100 μS/cm). The dominant source of instability for the high-conductivity solutions (***κ*** ≥ 500 μS/cm) may be either leaching (serum bottles) or evaporation (screw-cap bottles), depending on container type and size. Both leaching and evaporation increase proportionately with time of storage, up to the 2 years studied here.

The best package for the highest accuracy standards is the one in which the solution has either no change in ***κ*** with time or a small change in ***κ*** that does not significantly affect the target uncertainty (Table 1). The high-conductivity solutions should be packaged in containers in which there was no change in ***κ*** with time: ampoules (≥ 500 μS/cm) or serum bottles (100 000 μS/cm, only). The low-conductivity solutions had large changes in ***κ*** with time (> 0.7 %) when packaged in any container. Thus, they can not be stored for any length of time for the highest accuracy work. If long-term storage is necessary for less accurate work, the low-conductivity standards should be packaged in containers that showed the smallest change in ***κ*** with time: screw-cap bottles (≤ 100 μS/cm) or ampoules (15 μS/cm and 100 μS/cm, only).

Solution packaged in serum bottles was much less stable than the other packaging types tested. The instability of solutions packaged in serum bottles has also been found to be somewhat different from batch-to-batch at the same nominal conductivity.

The screw-cap bottles and ampoules both work equally well for some solutions. In these cases, convenience and cost should also be considered to determine which package is the best choice. The bottles are much more convenient in terms of filling, capping, and opening to make a measurement. Although the bottles are easy to ship, they can leak. The ampoules require much more time to fill and seal for the same amount of solution (10 times more ampoules than bottles would be needed). Although ampoules are easy to open to make a measurement, it may be necessary to open as many as 6 to obtain sufficient solution to make one measurement. The ampoules also cost more in terms of (1) original cost of the ampoule, (2) labor, due to the amount of time required for ampouling, and (3) shipping, due to their fragility. Therefore, in cases where the given solution packaged in ampoules or screw-cap bottles will have the same stability (e.g., 15 μS/cm and 100 μS/cm for short-term and long-term storage; 500 μS/cm for short-term storage), screw-cap bottles are the best choice.

Considering all of the applicable factors, cost, convenience, and stability (as discussed previously), the following recommendations are made for packaging of electrolytic conductivity standards: 5 μS/cm to 100 μS/cm in screw-cap bottles and 500 μS/cm to 100 000 μS/cm in ampoules. For conductivity values between 100 μS/cm and 500 μS/cm, the packaging type should be tested. The optimal point to switch from ampoules to screw-cap bottles occurs at a ***κ*** value between 100 μS/cm and 500 μS/cm but its exact value was not more thoroughly examined.

When examining other types of glass containers, the conductivity and mass change of the solution in the container should be examined to assess the stability of the solution in the given packaging type. The container should be air tight to eliminate evaporation. The glass used for the container should have the lowest possible concentration of leachable species, especially sodium, to minimize the effects of leaching. Leaching from glass containers can also be minimized by using a container which has a relatively large volume to surface area ratio.

Plastic containers are presently being studied. The results will be presented in future work.

## Figures and Tables

**Table 1:** Solution preparation

Nominal *κ*/(μS/cm)	Target uncertainty^a^/(%)	Screw-cap bottles	Packaging type Serum bottles	Ampoules	Approximate *m*_KCl_ per kg of solution/(g)
5	2	✔		✔	0.0053
15	0.7	✔	✔	✔	0.017
100	0.2	✔	✔	✔	0.050
500	0.07		✔	✔	0.25
1 000	0.07	✔	✔	✔	0.53
100 000	0.07		✔	✔	63

a Relative expanded uncertainty at the 95 % confidence interval.

**Table 2:** Compositions of glass types used for each container

Component	% Composition as provided by the manufacturer		
	Screw-capped bottles^a^	Serum bottles^b^	Ampoules^b^
SiO_2_	80.6	69.5	81
B_2_O_3_	13.0	10.4	13
Al_2_O_3_	2.3	5.5	2
Na_2_O + K_2_O	4.1	10.0	4.2
CaO + MgO		1.4	<0.02
BaO		2.5	<0.1
ZnO		0.5	
Minors			
(F, MnO_2_, Fe_2_O_3_, Li_2_O, CeO_2_)		0.3	

a See Ref. [11].

b See Ref. [12].

**Table 3:** Standard deviation, *s*^a^, at time, *t*^b^, for solutions packaged in screw-capped bottles

5 μS/cm		15 μS/cm		10 μS/cm		1000 μS/cm	
*t*	*s*	*t*	*s*	*t*	*s*	*t*	*s*
0	0.10	0	0.02	0	0.03	0	0.01
0.5	0.06	1.2	0.09	0.5	0.02	0.5	0.01
5.7	1.4	19.5	0.65	5.7	0.20	3.0	0.03
12.4	0.16			9.9	0.24	6.8	0.05
				12.4	0.09	12.6	0.07
						14.9	0.04
						18.8	0.10
						24.6	0.11

a Standard deviation has units of % relative.

b Time has units of months.

**Table 4:** Standard deviation, *s*^a^, at time, *t*^b^, for solutions packaged in serum bottles

15 μS/cm		100 μS/cm		500 μS/cm		1000 μS/cm		100 000 μS/cm	
*t*	*s*	*t*	*s*	*t*	*s*	*t*	*s*	*t*	*s*
0	0.04	0	0.25	0	0.03	0	0.00	0	0.01
1.0	0.59	9.1	2.7	4.4	0.02	0.6	0.01	3.6	0.00
2.7	2.3	12.5	1.2	8.4	0.51	3.9	0.01	8.1	0.01
6.4	3.0	18.6	11	12.6	1.3	6.0	0.13	12.6	0.01
9.9	4.5			15.6	2.0	12.1	0.27	18.0	0.04
13.1	4.6			20.5	2.9	14.0	0.76	24.2	0.01
18.1	6.1			24.0	3.3				
25.0	6.1								
30.1	2.6								

a Standard deviation has units of % relative.

b Time has units of months.

**Table 5:** Standard deviation, *s*^a^, at time, *t*^b^, for solutions packaged in ampoules

5 μS/cm		15 μS/cm		100 μS/cm		500 μS/cm		1000 μS/cm		100 000 μS/cm	
*t*	*s*	*t*	*s*	*t*	*s*	*t*	*s*	*t*	*s*	*t*	*s*
0.0	0.43	0.0	0.21	0.0	0.06	0.0	0.02	0.0	0.01	0.0	0.01
4.1	0.49	4.7	0.27	4.0	0.17	1.2	0.02	1.1	0.01	3.8	0.01
7.7	0.99	8.1	0.54	8.3	0.17	3.2	0.02	6.0	0.01	7.9	0.01
11.8	1.1	12.2	0.33	12.0	0.18	6.1	0.03	12.0	0.02	12.0	0.01
16.0	0.67	16.1	0.51	15.9	0.13	12.0	0.04	17.8	0.02		
19.9	1.1	19.9	0.40	20.1	0.10			25.1	0.05		

a Standard deviation has units of % relative.

b Time has units of months.

## Footnotes

1 Certain commercial equipment, instruments, or materials are identified in this paper to foster understanding. Such identification does not imply recommendation or endorsement by the National Institute of Standards and Technology nor does it imply that the materials or equipment identified are necessarily the best available for the purpose.
